# Community Trait Responses of Three Dominant Macrophytes to Variations in Flooding During 2011–2019 in a Yangtze River-Connected Floodplain Wetland (Dongting Lake, China)

**DOI:** 10.3389/fpls.2021.604677

**Published:** 2021-05-28

**Authors:** Ying Huang, Xin-Sheng Chen, Feng Li, Zhi-Yong Hou, Xu Li, Jing Zeng, Zheng-Miao Deng, Ye-Ai Zou, Yong-Hong Xie

**Affiliations:** ^1^Key Laboratory of Agro-ecological Processes in Subtropical Region, The Chinese Academy of Sciences, Changsha, China; ^2^Dongting Lake Station for Wetland Ecosystem Research, Institute of Subtropical Agriculture, The Chinese Academy of Sciences, Changsha, China; ^3^University of Chinese Academy of Sciences, College of Resources and Environment, Beijing, China

**Keywords:** freshwater wetland, emergent macrophyte, flood rising time, flood recession time, inundation duration

## Abstract

In lacustrine wetlands connected to rivers, the changes in flood regimes caused by hydrological projects lead to changes in the community traits of dominant macrophytes and, consequently, influence the structure and function of wetland vegetation. However, community trait responses of macrophytes to the timing and duration of flood disturbance have been rarely quantified. In 2011–2019, we investigated plant species diversity, density, and biomass in three dominant macrophyte communities (*Carex brevicuspis* C.B. Clarke, *Miscanthus sacchariflorus* (Maxim.) Hackel, and *Polygonum hydropiper* L.) through monthly field surveys in Dongting Lake wetlands. Partial least squares regressions were used to analyze how the variations in hydrological regimes affected plant community traits. Apparent inter-annual fluctuations in plant community traits were detected during 2011–2019. The species richness and Shannon index of diversity of *Miscanthus* and *Polygonum* communities increased, whereas the Shannon index of diversity of *Carex* community decreased. Variation in flooding had a greater effect on *Polygonum* and *Carex* community traits than on *Miscanthus* community traits. Flooding disturbed all plant communities, especially when the duration and timing varied. Shorter inundation periods caused the biomass of *Miscanthus* community to decline, and that of *Carex* and *Polygonum* communities to increase. Earlier flood recession caused the species richness and Shannon index of diversity of *Polygonum* and *Miscanthus* community to increase, and those of *Carex* community to decrease. These findings imply that shorter inundation durations and earlier flood recession generated by the operation of the Three Gorges Dam have changed the macrophyte growth pattern.

## Introduction

Wetlands have the hydrological characteristics of alternating between land and water, and dry and wet (Zhang et al., 2020a,b). Macrophytes are integral components of wetlandccc ecosystems (Bakker et al., 2012; Fares et al., 2020) and are often subjected to periodic flood stress conditions (Chen et al., 2015a; Sousa et al., 2020). Macrophytes have evolved to adapt to hydrological fluctuations and exhibit various survival strategies, such as modified life history traits and the regulation of physiological processes (Hough-Snee et al., 2015). However, the changes in flooding regime influence the growth and developmental activities of macrophytes, and understanding how flood regime affects macrophyte can assist in managing vegetation restoration and in maintaining wetland function and diversity (Jyothi and Sureshkumar, 2018).

Flood regime can be organized into five categories: timing, duration, magnitude, frequency, and change rate of flooding (Richter et al., 1996; Tiner, 2009). Among them, timing and duration of flooding are considered as important components (Mu et al., 2020; Yang et al., 2020). Timing is organized into start time and end time of flood (Jing et al., 2017). The timing and duration of flooding affect plant species composition and productivity in wetlands by stimulating or inhibiting germination and the subsequent growth, by modifying oxygen availability and limiting the light intensity and nutrients (Coops et al., 2003; Liu et al., 2017; Yao et al., 2020). Several studies have described the effect of flood regime on plants in wetlands (Keddy, 1983; Chen et al., 2015b; Lawson et al., 2015; Gao et al., 2019). Prolonged inundation causes the species richness of intermittent wetland seed bank to decline, whereas short floods increase biomass and species richness of macrophyte communities (Casanova and Brock, 2000). *Carex* biomass reportedly decreases with extended periods of inundation (Guan et al., 2014), whereas *Carex* distribution areas decrease under earlier or delayed flood recession (Guan et al., 2016). In comparison, studies on the combined effects of quantified timing and duration of flooding on the traits of vegetation communities are rare.

Hydrological regimes in numerous basins have shifted substantially under the influence of anthropogenic activities, especially hydrological projects (Gao et al., 2017). For example, the Three Gorges Dam Project is the largest water control project in the world and is located in the upper reaches of the Yangtze River. The Dongting Lake wetlands, the second largest freshwater lake in China, have been experiencing large hydrological changes since the operation of the Three Gorges Dam (Lai et al., 2016; Gao et al., 2017). The discharge from Yangtze River into Dongting Lake has decreased; the water level of Dongting Lake and the duration of high water level have also decreased (Xie et al., 2014; Gao et al., 2019; Yang et al., 2020). With the fluctuations in water level in space and time, the development of macrophyte community and patterns of macrophyte zonation in the wetland changes annually and seasonally (Guan et al., 2016; Li et al., 2018; Yu et al., 2018). Nevertheless, it is difficult to predict the composition of the plant communities in relation to flood regime (Xie et al., 2014; Hu et al., 2015).

Here, we investigated the dominant macrophyte communities (*Carex brevicuspis* C.B. Clarke, *Miscanthus sacchariflorus* (Maxim.) Hackel, and *Polygonum hydropiper* L.) in Dongting Lake over nine consecutive years. Field survey data were collected monthly, whereas hydrological data were collected daily. Two hypotheses were tested. Delayed rise of floods and earlier flood recession might prolong the season of plant growth and development, whereas the earlier rise of floods and delayed recession of floods might shorten the growing season of macrophyte communities. Therefore, we firstly hypothesized that the earlier rise of floods and delayed recession of floods would cause macrophyte community traits (such as biomass and species richness) to decline, whereas delayed rise in floods and earlier recession in floods would cause macrophyte community traits to increase. We also hypothesized that the variations in flooding would have a greater effect on the community traits of *Carex* and *Polygonum* than those of *Miscanthus*, because the timing of rise and fall of water levels appears to influence *C. brevicuspis* and *P. hydropiper* more than *M. sacchariflorus*, because the former occurs at lower elevations than the latter.

## Materials and Methods

### Study Area

Dongting Lake (28°30'–30°20'N; 111°40'–113°10'E) is located in the middle reaches of the Yangtze River (Yu et al., 2018). Dongting Lake is composed of three sub-basins, namely, the western, southern, and eastern sub-basins of Dongting Lake. The lake is fed by three inlets (the Songzi, Taiping, and Ouchi) and four major tributaries (the Xiang, Zi, Yuan, and Li rivers). The water eventually drains back into the Yangtze River from the Chenglingji outlet. It is a seasonal, flood-channel lake and is an important part of the Yangtze River ecosystem (Wu et al., 2019). The mean annual temperature is 16.8°C, and annual precipitation is 1,382 mm, with more than 60% falling between April and August. For decades, the major changes in Dongting Lake wetlands were vegetation expansion and water area shrinkage (Yang et al., 2020). Dongting Lake had lost almost two-thirds of its total area in the past century (Du et al., 2001).

East Dongting Lake is the largest water basin in the region. In 1992, the East Dongting Lake Nature Reserve was included in the list of the Ramsar Convention on Wetlands of International Importance. The wetlands in East Dongting Lake are characterized by large seasonal water-level fluctuations (up to 15 m). The flooding and nonflooding periods generally range from June to October and from November to May of the next year, respectively (Zhang et al., 2020a,b). Plant zonation along an elevation gradient is a common phenomenon (Xie and Chen, 2008). Currently, *C. brevicuspis* is mainly distributed at elevations of 22–27 m (Tang et al., 2013). *M. sacchariflorus* is mostly distributed at elevations above 27 m, and shelter forests grow above 30 m elevation (Hou et al., 2016). The three macrophyte communities, especially *Carex* community, are important for providing habitats and foods for migratory waterfowls in the floodplain (Zhang et al., 2020a,b).

### Plant Communities in the Study Area

*Carex brevicuspis* (*Cyperaceae*) has overlapping leaf sheaths, and individuals are typically 20–55 cm tall (Chen et al., 2015b). *P. hydropiper* (Polygonaceae) is an annual herb with branched stems that are normally 40–70 cm long, and it often co-occurs with *Carex* species (Chen et al., 2015a). The culms of *M. sacchariflorus* (Poaceae) are slender, erect, and 100–500 cm long (Chen et al., 2019). These three species often form mono-dominant communities along elevational gradients in the Dongting Lake wetlands. From the water’s edge to the uplands of Dongting Lake wetlands, the general plant zonation pattern consists of *C. brevicuspis* communities (hereinafter referred to as *Carex*); *P. hydropiper* communities (*Polygonum*) generally embedded within the *Carex* zone; and *M. sacchariflorus* communities (*Miscanthus*; Xie and Chen, 2008).

During the flooding season (usually June–October), *C. brevicuspis* is completely submerged, and the aboveground shoots senesce. Shoots emerge immediately after flooding and grow into a standing crop before January (Chen et al., 2015b). Most of the aboveground shoots of *M. sacchariflorus* and some of the aboveground shoots of *P. hydropiper* can survive flooding, and flower and fruit after this period (Voesenek et al., 2006; Qin et al., 2013).

### Data Collection

#### Field Sampling

**Figure 1:**
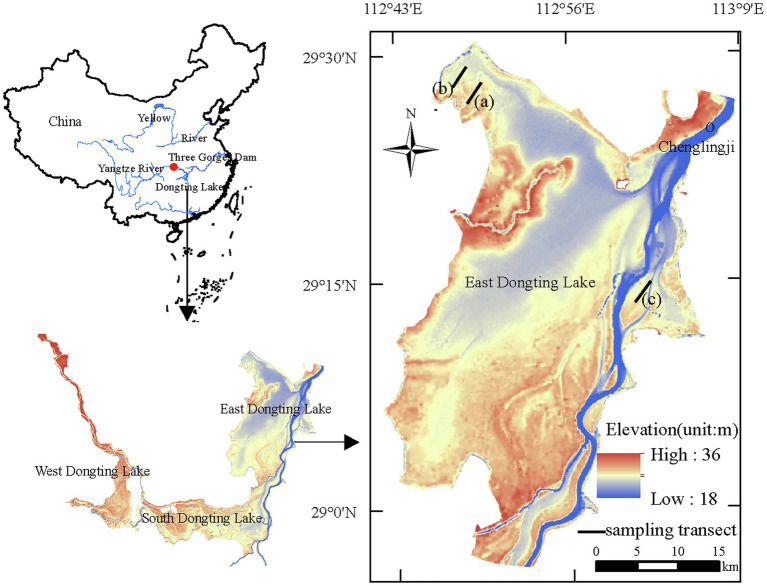
Locations of the sampling sites in East Dongting Lake, China. (a) *Carex* community, (b) *Miscanthus* community, and (c) *Polygonum* community.

Three lake shore areas harboring the three plant communities were selected as study sites (Figure 1). In each site, a transect (1,000 m × 100 m) was established parallel to the lake shore through the middle of the plant communities. In each transect, five quadrats (1 m × 1 m) were established every 50 m, with a total of 15 quadrats. The corners of each quadrat were marked by hammering durable plastic tubes into the soil. During the surveys, species composition, density, plant height, and plant cover in the quadrats were recorded. The position of the study sites was delineated using a hand-held global positioning system device (UniStrong Odin Series, Beijing, China). Plant community characteristics within the quadrats were surveyed monthly when wetlands were exposed during 2011–2019.

#### Plant Community Data

Photographs of unrecognized plant species were taken, and specimens were obtained. The specimens were transferred to the laboratory for identification. Aboveground shoots within the quadrats were also clipped and transported to the laboratory. Aboveground shoots were dried in an oven at 60°C for 48 h to obtain dry weight. Plant biomass was defined as the dry biomass of the aboveground shoots. Species diversity was expressed using the Shannon–Wiener diversity index (Khedr and El-Demerdash, 1997) and calculated using the following formula:

(1) $$H=-\sum_{i=1}^{S}P_{i}\ln\left(P_{i}\right)$$

where *S* is the number of species within a quadrat and $P_{i}=n_{i}/N$, where *n_i_* is the number of individuals in the *i*th species, and *N* is the plant density within a quadrat.

#### Environmental Variables

Daily water levels were recorded at 08:00 h in the Chenglingji Hydrological Gauging Station during 2011–2019 and were used to represent the hydrological regimes of East Dongting Lake. These records have been used extensively in previous hydrological studies in that section of the lake (Chen et al., 2015a). Precipitation and flow rate data of Chenglingji were obtained from the Hydrology and Water Resources Survey Bureau of Hunan Province.^1^

The hydrological regimes of macrophytes in East Dongting Lake wetlands are highly correlated with ground surface elevation (Urban, 2005; Xie et al., 2014). In theory, macrophytes are considered submerged when water levels exceed a certain elevation. Here, the elevation in each sampling site was derived from a 30-m spatial resolution digital elevation model of Dongting Lake wetlands in 2009. The model was obtained from Geospatial Data Cloud.^2^

Among various hydrological parameters, flood rising time, flood recession time, and inundation duration were considered as important factors that influence the growth of macrophytes (Liu et al., 2019; Torso et al., 2020). Hydrological variables such as flood rising time, flood recession time, and inundation duration at different sampling sites were calculated based on daily water-level data and elevation data. Water depth was measured as the distance from the water surface to the ground surface and was calculated as follows:

(2) WD=WL−E

Flood rising time was calculated as follows:

(3) $$WR1=\min\left\{t|WD_{t}>0\right\}$$

Flood recession time was calculated as follows:

(4) $$WR2=\max\left\{t|WD_{t}>0\right\}$$

Inundation duration was calculated as follows:

(5) $$ID=\sum_{WD>0}^{n}I_{WD}$$

where *WL* is the water level in the Chenglingji Hydrological Station and *E* is the elevation; *t* is the day of year; *WD_t_* is the water depth at *t*; $I_{WD}$ is the number of days when *WD* > 0; and *n* is the number of days within a year.

### Statistical Methods

Plant community traits such biomass accumulation and species diversity are influenced by water levels (Silva et al., 2013; Zhang et al., 2020a,b), and the peak values reflect the community traits in a certain year (Chen et al., 2015a; Mu et al., 2020). Therefore, the maximum plant community traits before and after flooding were selected. There is always a time lag between hydrological changes and vegetation responses (Hou et al., 2019). Therefore, before analyzing the effect of hydrological changes on plant community traits, sum of the maximum value of the plant community after flooding and the maximum value before flooding was considered as a dependent variable, to control the influence of plant community traits before flooding.

The effects of hydrological regimes on plant community traits were analyzed using partial least squares regression (PLS). PLS is to study regression modeling of multiple dependent variables to multiple independent variables. This approach addresses potential challenges, such as the non-normal distribution of data, factor score indeterminacy, and model type where it is not recognized. Hydrological regimes include many factors such as timing of flood rise, timing of flood recession, and inundation duration. Because the correlations among variables were relatively high, and the sample size was low, PLS was used to analyze the effect of flood regimes on plant community traits.

**Figure 2:**
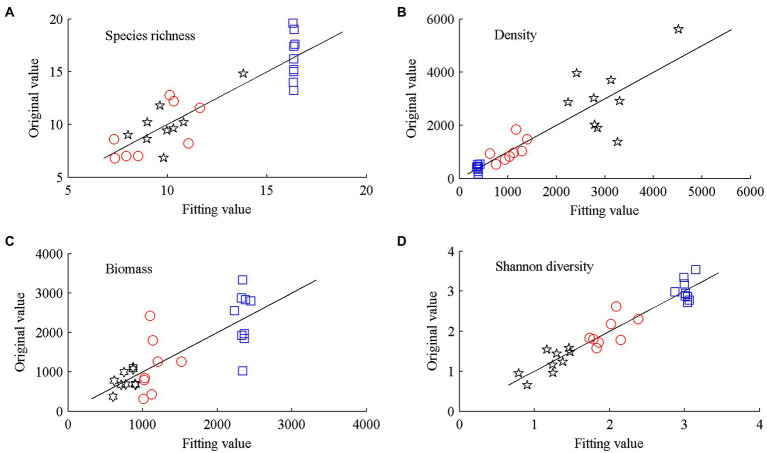
Fitting effects of the PLS models of traits of the three plant communities (black five-pointed star—*Carex* community, blue square—*Miscanthus* community, and red circle—*Polygonum* community). (**A**) Species richness; (**B**) Density; (**C**) Biomass; and (**D**) Shannon diversity.

The independent variables in the PLS model were the hydrological regimes of each community type, whereas the dependent variables were the sum values of plant community traits before and after flooding. The differences between the values fitted using the equation and the actual values were negligible (Figure 2). The fitting effect of the PLS model was largely accurate (*F* < 0.05). All statistical analyses were performed using R package (version 3.5.1).

## Results

### Changes in the Hydrological Regime in Plant Stands

**Figure 3:**
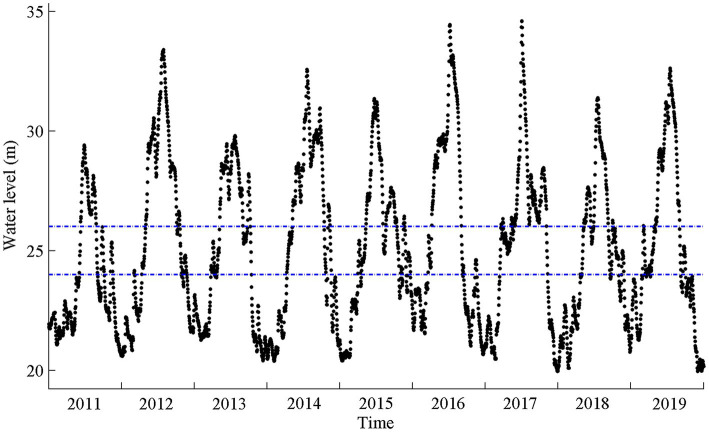
Daily water levels in the Chenglingji Hydrological Station during 2011–2019. The blue dashed lines that are parallel to X-axis indicate the ranges of elevations at the field sampling points.

The amplitude of intra-annual water levels at Chenglingji Hydrological Station was lower in 2011 than in 2012, 2016, and 2017 (Figure 3). During 2011–2019, the average annual flow rate in Chenglingji Hydrological Station was the largest in 2016 (9,865 m^3^/s), followed by that in 2017 (8,689 m^3^/s), and the smallest in 2011 (4,721 m^3^/s). The annual precipitation at Yueyang Meteorological Station was the largest in 2015 (1725.3 mm), followed by that in 2017 (1650.7 mm), and the smallest in 2011 (1650.7 mm).

**Figure 4:**
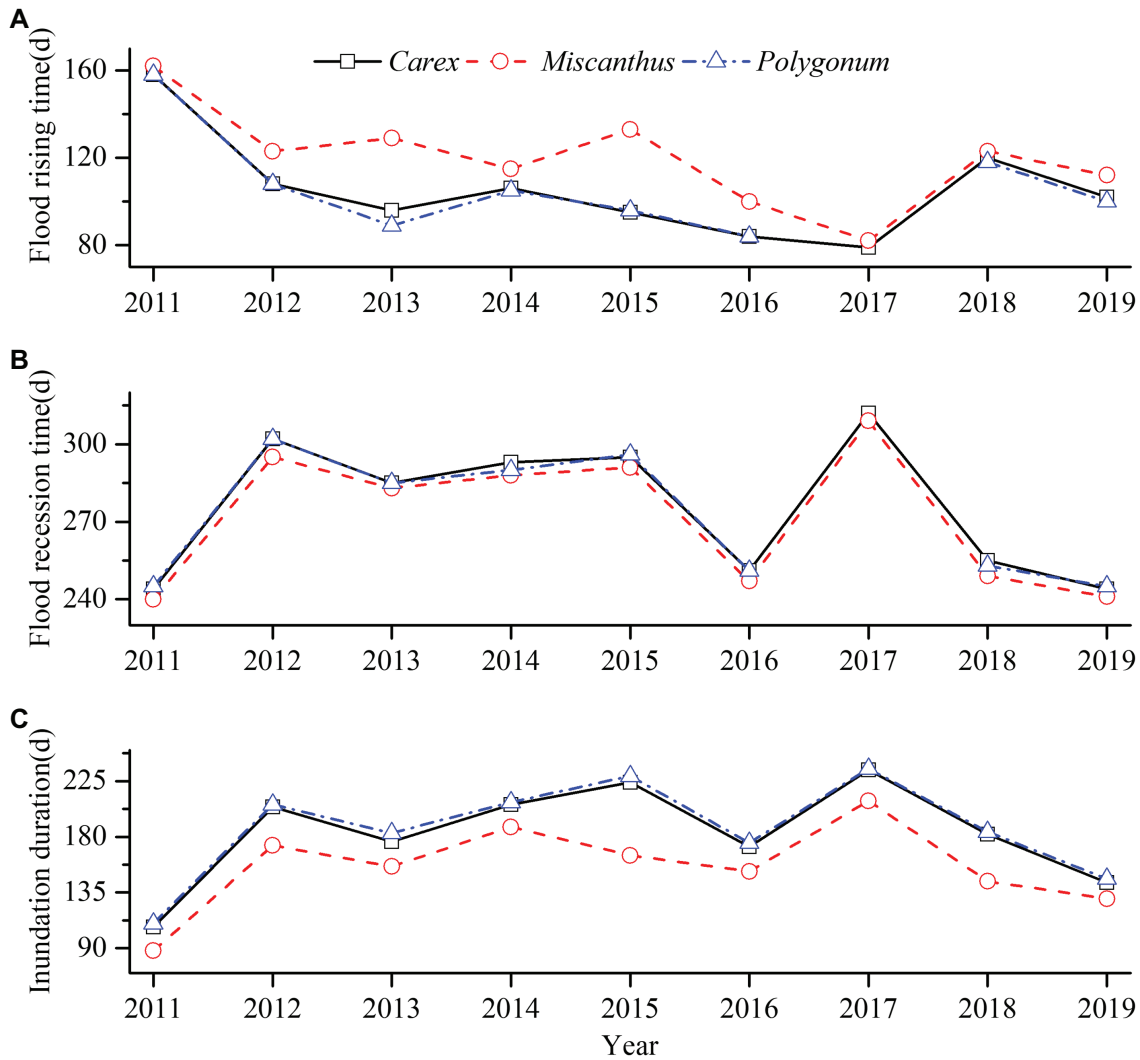
Flood rising time (**A**), flood recession time (**B**), and inundation duration (**C**) in the three plant communities of Dongting Lake wetlands (black solid line—*Carex* community, red dash line—*Miscanthus* community, and blue dash dot line*—Polygonum* community) during 2011–2019.

Flood rising time, flood recession time, and inundation duration noticeably varied during 2011–2019 (Figure 4). The water level increased in early June of 2011, but increased in early March of 2017. In 2011, 2016, and 2018, the water level receded in early September, whereas it receded in early November in 2012 and 2017. Inundation duration was shorter in 2011 than in 2017. The flood rising time in all the three studied plant communities shifted to an earlier date, whereas the inundation duration exhibited a parabolic trajectory, finally shortening (Table 1 and Figure 4). There was a negative correlation between flood rising time and inundation duration, and a significant positive correlation between flood recession time and inundation duration (Figure 4).

**Table 1:** Summary of regression analysis of flood regime in the three plant communities (*Carex brevicuspis*, *Miscanthus sacchariflorus*, and *Polygonum hydropiper*) during 2011–2019.

Community type		*K*_1_	*K*_2_	*R*^2^	*F*
*Carex*	*FR1*	0.0219^*^	−0.253^*^	0.6625	5.888^*^
	*ID*	−0.030^*^	0.320^*^	0.6391	5.312^*^
*Miscanthus*	*FR1*	−	−0.042^*^	0.3668	4.054^*^
	*ID*	−0.029^*^	0.315^*^	0.5996	4.493^*^
*Polygonum*	*FR1*	0.020^*^	−0.232^*^	0.6106	3.921^*^
	*ID*	−0.0282^*^	0.295^*^	0.6462	4.566^*^

Asterisks denote significant levels

* *p < 0.05*.

*K*_1_, quadratic coefficient; *K*_2_, first-order coefficient; *FR1*, flood rising time; *ID*, inundation duration.

### Changes in Plant Community Traits

**Figure 5:**
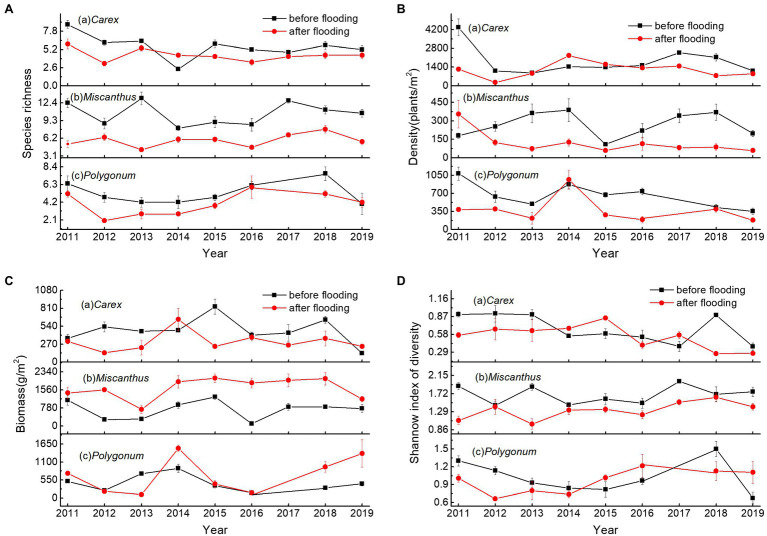
Maximum species richness (**A**), density (**B**), biomass (**C**), and Shannon index of diversity (D) values before and after flooding in the three plant communities in Dongting Lake wetlands during 2011–2019.

In 2011–2019, there were clear inter-annual fluctuations in the plant community characteristics at the three sites (Figure 5). Before flooding, the species richness of *Polygonum* community exhibited a parabolic trajectory, finally increasing, whereas the density declined (Table 2 and Figure 5). The Shannon index of diversity of *Carex* community also decreased (Figure 5). The density and biomass of *Miscanthus* community increased in two phases (Figure 5).

**Table 2:** Summary of regression analysis of *C. brevicuspis*, *M. sacchariflorus*, and *P. hydropiper* community traits (species richness, density, biomass, and Shannon index of diversity) from 2011 to 2019.

Time	Type	Traits	*K*_1_	*K*_2_	*R*^2^	*F*
Before flooding	*Polygonum*	*SR*	0.029^*^	−0.224^*^	0.6413	10.6^*^
		*D*	–	−0.097^*^	0.5359	6.928^*^
After flooding	*Carex*	*SW*	–	−0.032^*^	0.4593	5.946^*^
	*Miscanthus*	*D*	–	−0.135^*^	0.4588	5.934^*^
		*SW*	–	0.06^*^	0.4825	5.593^*^

Asterisks denote significant levels

* *p < 0.05*.

*SR*, species richness; *SW*, Shannon index of diversity; *D*, density; and *B*, biomass; *K*_1_, quadratic coefficient; *K*_2_, first-order coefficient.

After flooding, *Carex* biomass decreased, whereas *Miscanthus* biomass increased (Figure 5). The species richness and Shannon index of diversity of *Miscanthus* and *Polygonum* communities increased, whereas the Shannon index of diversity of *Carex* community decreased (Table 2 and Figure 5).

### Trait Dynamics of the Plant Communities Relative to the Hydrological Changes in Plant Stands

**Figure 6:**
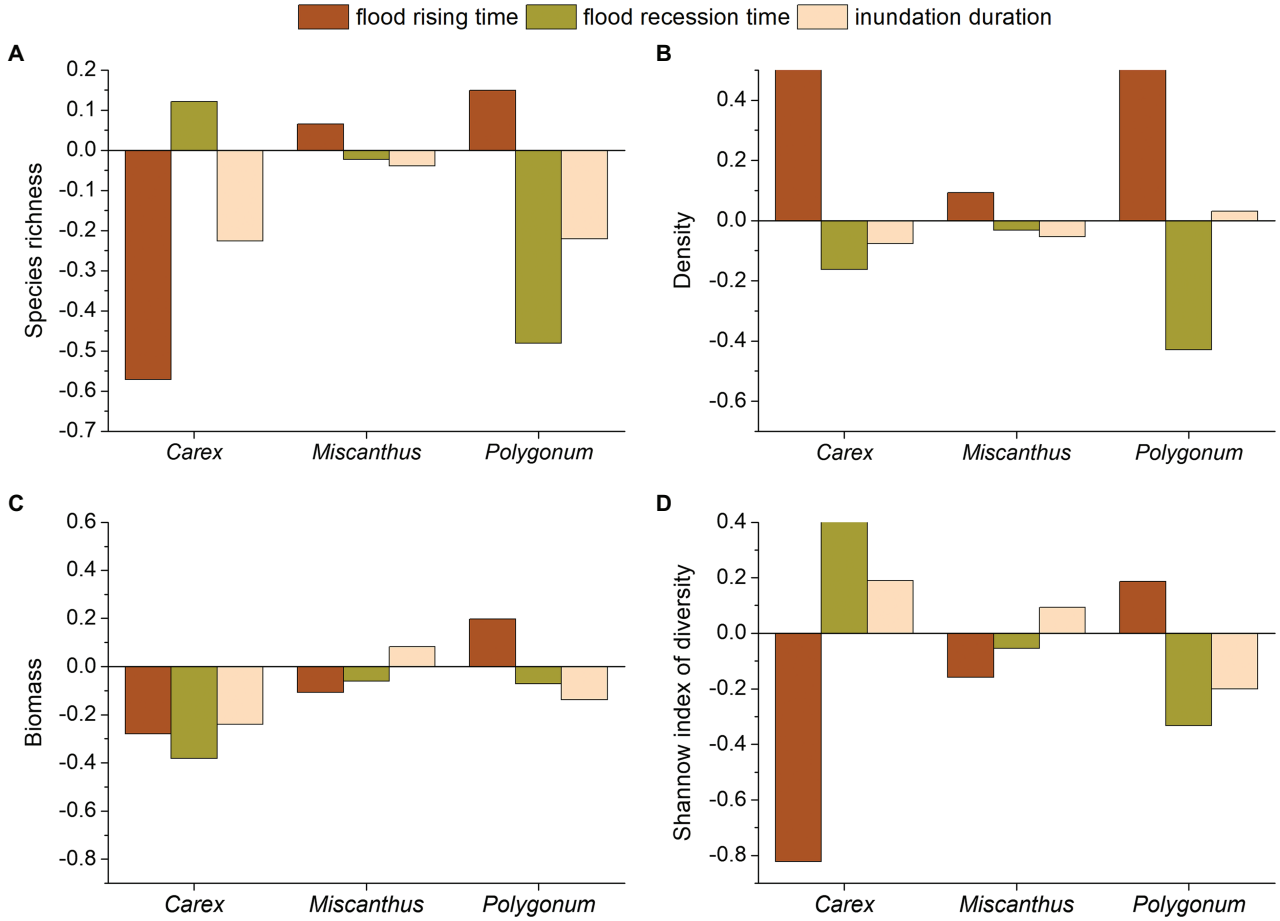
Marginal effects of each hydrological variable (including flood rising time, flood recession time, and inundation duration) on the community traits (species richness (**A**), density (**B**), biomass (**C**), and Shannon index of diversity (**D**) of the three plant communities) of three macrophytes in Dongting Lake wetlands.

Based on species richness and Shannon index of diversity, variations in flooding had a greater effect on *Polygonum* and *Carex* communities than on *Miscanthus* community (Figure 6). Hydrological variables influenced *Polygonum* community in the following order: flood recession time > flood rising time > inundation duration. In addition, shorter inundation, earlier flood recession, or delayed flood rise significantly increased the species richness and Shannon index of diversity of *Polygonum*, whereas delayed flood rise and advanced flood recession caused the species richness and Shannon index of diversity of *Carex* community to decrease. Flood recession time had a low capacity to explain the species richness and Shannon index of diversity of *Miscanthus* (Table 3 and Figure 6).

**Table 3:** Summary of partial least square analysis of the effect of hydrological regimes on *C. brevicuspis*, *M. sacchariflorus*, and *P. hydropiper* community traits (species richness, density, biomass, and Shannon index of diversity).

*Variable*	*Carex* community			
	*SR*	*SW*	*B*	*D*
*Flood rising time*	−0.055^*^	−0.011^*^	−2.873^*^	29.239^*^
*Flood recession time*	0.002^*^	0.007^*^	−3.380^*^	−7.726^*^
*Inundation duration*	−0.013^*^	0.002^*^	−1.429^*^	−2.473^*^
*Variables*	*Miscanthus* community			
	*SR*	*SW*	*B*	*D*
*Flood rising time*	−0.007^*^	−0.002^*^	−1.518^*^	0.496^*^
*Flood recession time*	*−*0.002^*^	−0.005^*^	−0.429^*^	−0.140^*^
*Inundation duration*	0.002^*^	0.007^*^	0.571^*^	−0.187^*^
*Variables*	*Polygonum* community			
	*SR*	*SW*	*B*	*D*
*Flood rising time*	0.007^*^	0.003^*^	6.031^*^	0.003^*^
*Flood recession time*	−0.049^*^	−0.005^*^	−0.067^*^	−0.005^*^
*Inundation duration*	−0.014^*^	−0.002^*^	−1.007^*^	−0.002^*^

Asterisks denote significant levels

* *p < 0.05*.

*SR*, species richness; *SW*, Shannon index of diversity; *D*, density; and *B*, biomass.

Inundation duration had a low capacity to explain the density of plant communities. Hydrological variables influenced *Carex* and *Polygonum* densities in the following order: flood rising time > flood recession time > inundation duration. In addition, early flood rise and delayed flood recession caused the density of *Carex* and *Polygonum* communities to decline (Table 3 and Figure 6).

Variation in flooding had a greater effect on the biomass of *Carex* and *Polygonum* than on *Miscanthus* (Figure 6). Hydrological variables influenced *Miscanthus* in the following order: flood rising time > inundation duration > flood recession time. The hydrological variables influenced *Carex* biomass in the following order: flood recession time > inundation duration > flood rising time. Shorter inundation duration and advanced flood recession caused the biomass of *Carex* to increase (Table 3 and Figure 6).

## Discussion

### Influence of Different Hydrological Variables on Plant Community Traits

Flood rising time had the greatest influence on *Polygonum* and *Miscanthus* biomasses, whereas flood recession time was a key factor affecting the biomass of *Carex*. *Carex brevicuspis* is the dominant species in *Carex* community, which has two growing periods every year. *Carex brevicuspis* basically completes its first growing season before the annual flood and resprouts after the flood period, completing its second growing season in December (Chen et al., 2015b). The flood recession time affects the resprouting rate of *C. brevicuspis* and the duration of the second growing season. Therefore, flood disturbance had a greater influence on *Carex* community after the flood than before the flood. However, the life history of *M. sacchariflorus* and *P. hydropiper* differs from that of *C. brevicuspis*. These two species survive flooding through morphological and physiological mechanisms, such as shoot elongation and low metabolism (Chen et al., 2019). After flooding, *M. sacchariflorus* and *P. hydropiper* resume growth, and they flower and fruit in November (Deng et al., 2013). Therefore, flood recession time is a key factor affecting the biomass of *Carex*, whereas flood rising time was the most influential factor on *Polygonum* and *Miscanthus* biomasses.

Early flood rising and early flood recession would increase the Shannon index of diversity of *Miscanthus*, whereas delayed flood rising and early flood recession would decrease the Shannon index of diversity of *Carex*. When flood recession is advanced, the growing period of *Carex* community is extended, and it forms a closed community (with high coverage and high density), hindering the survival of other plant species, resulting in a decrease in species richness and diversity (Pan et al., 2006; Li et al., 2013; Wang et al., 2019). However, *Miscanthus* community is relatively tall and can form stratification, providing a space for the growth of other short herbs, resulting in higher species richness and diversity (Qin et al., 2013; Wang et al., 2016; Qi et al., 2021).

Early flood rising and early flood recession would increase the biomass of *Carex* and *Miscanthus*, whereas early flood wrising and delayed flood recession would decrease the biomass of *Polygonum*. Previous studies have pointed out that delayed flood recession would significantly decrease the intrinsic and maximum growth rates of *Carex*, resulting in significantly reduced biomass (Guan et al., 2014). Furthermore, when the flood recession is advanced, the growing period of the macrophytes is extended. This phenomenon might facilitate the growth of macrophytes, resulting in a greater biomass of macrophyte community (Qin et al., 2013).

Delayed flood rising and early flood recession time are likely to increase the density of *Carex*, *Miscanthus*, and *Polygonum* communities. The flooding periods in Dongting Lake generally range from June to October. Shallow submersion only suppresses macrophytes tillering and causes the colonized area to decrease (Chen et al., 2015b, 2019). Delayed flood rising decreases the chances of complete submergence of macrophytes. Furthermore, macrophytes are likely to have accumulated some storage material in the roots (Yuan et al., 2019; Song et al., 2021). Under such conditions, these macrophytes can survive the flood for several weeks, but they suffer severe damage (Li et al., 2013). Early flood recession time helps plant recover from flood damage. The results of the current study are similar to those of Klimešová (1994), who observed that the growth of *Phalaris arundinacea* L. and *Urtica dioica* L. under delayed flood rising was better than that of plants under early flood rising.

Moreover, prolonged inundation decreased the biomass of *Carex* and *Polygonum* communities to decrease, but increased the biomass of *Miscanthus*. Specifically, following prolonged inundation in 2012 and 2015 (Figure 4), the biomass of *Carex* and *Polygonum* communities significantly decreased compared with that before flooding, whereas the biomass of *Miscanthus* community increased significantly after flooding (Figure 5). Prolonged inundation would significantly decrease the intrinsic growth rate of *Carex*, but increasing the time required to reach the maximum growth rate. This phenomenon would significantly reduce shoot density and aboveground biomass. When only partially inundated, the growth of *Miscanthus* may be enhanced through shoot elongation to escape the flood. This phenomenon would cause aboveground biomass to increase. *Polygonum* was less abundant than *Miscanthus*; *Polygonum* often co-occurs with *Carex* species, which are sensitive to inundation duration. Prolonged inundation might shorten the plant growth and development season. The results of the current study are similar to those of Guan et al. (2014), who observed that prolonged inundation significantly lowered the biomass accumulation of *Carex*.

Overall, our first hypothesis was rejected; that is, early flood rising and delayed flood recession would decrease biomass and diversity, whereas delayed flood rising and early flood recession would increase biomass and diversity of macrophyte communities.

### Differed Responses Among Plant Communities to Hydrological Regimes

In lacustrine wetlands connected to rivers, fluvial disturbance in the form of water level fluctuations affect all resident plant communities, particularly in relation to the timing and duration of flood disturbance, but with varying frequency and amplitude. *Polygonum* and *Carex* communities were considerably more influenced by flooding than *Miscanthus* community, based on species richness, density, biomass, and Shannon index of diversity. This might be explained by the fact that *Polygonum* and *Carex* communities occupy lower elevations than *Miscanthus* community. In comparison with communities occurring at higher elevations, low-elevation communities are influenced by flooding for longer periods and at higher frequencies. Second, the height of *C. brevicuspis* and *P. hydropiper* is considerably shorter than that of *M. sacchariflorus*, which can grow to a height of 500 cm (Chen et al., 2015b). Consequently, *C. brevicuspis* and *P. hydropiper* are more vulnerable to flooding, whereas most *M. sacchariflorus* in *Miscanthus* community can survive the flooding season (Kettenring and Galatowitsch, 2007). The results of the current study are consistent with those of Chen et al. (2015b), who observed that *Carex* and *Polygonum* communities are more sensitive to the timing of disturbance than *Miscanthus* community based on belowground bud banks. Previous studies suggest that the closer to the margins of the key environmental gradient, the more sensitive the species are to habitat change (Heino and Mendoza, 2016; Lou et al., 2020).

Overall, our second hypothesis was supported, whereby flooding variability may have a greater effect on *Carex* and *Polygonum* community characteristics than on *Miscanthus* community.

### Implications for Vegetation Restoration and Management

The varying flood regimes led to varying plant community characteristics, which led to vegetation succession (Casanova and Brock, 2000). Our results demonstrated that shorter inundation durations and earlier flood recession generated by the operation of the Three Gorges Dam have caused the density of *Miscanthus* community to increase and the density of *Carex* community to decrease. Consequently, *Miscanthus* community is expanding, whereas *Carex* community is shrinking. *Carex* habitat provides an important food source for wintering migratory birds, with its loss potentially affecting the distribution and quantity of migratory birds (Zhang et al., 2020a,b). Such potential effects of flooding regimes should be taken into account when implementing any hydrological projects that could alter flooding regimes in Dongting Lake. The results of the present study could be used to predict the changes in macrophyte communities under altered hydrological conditions in other areas.

## Data Availability Statement

The raw data supporting the conclusions of this article will be made available by the authors, without undue reservation.

## Author Contributions

YH and X-SC wrote the manuscript and executed statistical analyses. X-SC and Y-HX designed the experiments and edited the manuscript. FL, Z-YH, JZ, XL, Z-MD, and Y-AZ contributed to data collection and interpretation. All authors contributed to the article and approved the submitted version.

### Conflict of Interest

The authors declare that the research was conducted in the absence of any commercial or financial relationships that could be construed as a potential conflict of interest.

## Footnotes

^1^http://www.hnwr.gov.cn/

^2^http://www.gscloud.cn/
